# Differential roles and regulation of the protein kinases PAK4, PAK5 and PAK6 in melanoma cells

**DOI:** 10.1042/BCJ20220184

**Published:** 2022-08-31

**Authors:** Gavuthami Murugesan, Alan R. Prescott, Rachel Toth, David G. Campbell, Claire M. Wells, Carol MacKintosh

**Affiliations:** 1 Division of Cell and Developmental Biology, University of Dundee, Dundee DD1 5EH, Scotland, U.K.; 2 Division of Cell Signalling and Immunology, University of Dundee, Dundee DD1 5EH, Scotland, U.K.; 3 MRC Protein Phosphorylation and Ubiquitylation Unit, School of Life Sciences, University of Dundee, Dundee DD1 5EH, Scotland, U.K.; 4 Division of Cancer Studies, King's College London, New Hunts House, Guy's Campus, London SE1 1UL, U.K.

**Keywords:** 14-3-3 proteins, evolutionary biology, phosphorylation/dephosphorylation, protein-serine-threonine kinases

## Abstract

The protein kinases PAK4, PAK5 and PAK6 comprise a family of ohnologues. In multiple cancers including melanomas *PAK5* most frequently carries non-synonymous mutations; *PAK6* and *PAK4* have fewer; and *PAK4* is often amplified. To help interpret these genomic data, initially we compared the cellular regulation of the sister kinases and their roles in melanoma cells. In common with many ohnologue protein kinases, PAK4, PAK5 and PAK6 each have two 14-3-3-binding phosphosites of which phosphoSer99 is conserved. PAK4 localises to the leading edge of cells in response to phorbol ester-stimulated binding of 14-3-3 to phosphoSer99 and phosphoSer181, which are phosphorylated by two different PKCs or PKDs. These phosphorylations of PAK4 are essential for its phorbol ester-stimulated phosphorylation of downstream substrates. In contrast, 14-3-3 interacts with PAK5 in response to phorbol ester-stimulated phosphorylation of Ser99 and epidermal growth factor-stimulated phosphorylation of Ser288; whereas PAK6 docks onto 14-3-3 and is prevented from localising to cell–cell junctions when Ser133 is phosphorylated in response to cAMP-elevating agents via PKA and insulin-like growth factor 1 via PKB/Akt. Silencing of PAK4 impairs viability, migration and invasive behaviour of melanoma cells carrying *BRAF^V600E^* or *NRAS^Q61K^* mutations. These defects are rescued by ectopic expression of PAK4, more so by a 14-3-3-binding deficient PAK4, and barely by PAK5 or PAK6. Together these genomic, biochemical and cellular data suggest that the oncogenic properties of PAK4 are regulated by PKC–PKD signalling in melanoma, while PAK5 and PAK6 are dispensable in this cancer.

## Introduction

Cancer is a disease of the genome — of chromosomal amplifications, losses, rearrangements, epigenetic modifications, and hundreds, sometimes thousands of small-scale mutations. Cancer is also a disease in which dysregulated signalling networks lock cancer cells into progressive programmes of metabolic change, proliferation, migration, and tissue invasion.

Major advances have been made in reconciling the genomic and cell signalling perspectives of cancers. Specific driver mutations have been identified in diverse genes, many of which boost cancer growth by activating the PI 3-kinase–PKB/Akt–mTOR and Ras–Raf–Mek–Erk–p90RSK signalling pathways [1]. For example, in most skin melanomas Erk signalling is driven by gain-of-function mutations in *BRAF* or *NRAS* [1–3].

Signalling by activated B-Raf and N-Ras is affected by wider signalling networks that influence malignant cell phenotypes. For example, PKC–PKD is often co-activated with Ras–Raf–Mek–Erk–p90RSK signalling in melanoma [4–8] and historically PKC has been considered oncogenic, stemming from the tumour-promoting actions of its activator phorbol ester. However, PKC inhibitors have failed as anti-tumour agents in clinical trials, many cancer-associated loss-of-function PKC mutations have been identified, and recent evidence indicates that PKC generally acts as a tumour suppressor. It is suggested that the tumour-promoting effects of phorbol esters could result from their promoting the chronic loss of PKC. The emerging consensus is that PKC potentially inactivates oncogenes and stabilises tumour suppressors [8–12].

Many components of PI 3-kinase–PKB/Akt–mTOR, Ras–Raf–Mek–Erk–p90RSK and PKC–PKD signalling, and their downstream effectors, exist in variant forms expressed by families of two, three or four ‘ohnologue’ genes that were generated by two rounds of whole genome duplication (2R-WGD) at the evolutionary origin of the vertebrates [13–16]. While most of the extra genes generated by the 2R-WGD have since been lost, those ohnologue gene families that still exist in modern-day vertebrates are highly enriched in signalling components such as receptors and protein kinases [16]. Ohnologues comprise only ∼25% of human genes, but 66% of protein kinases, including the Raf (A,B,C), Erk (1,2), p90RSK (1,2,3), conventional PKC (α,β,γ), novel PKC (δ,η and ε,θ) and PKD (1,2,3) families [14,17,18].

Interestingly, ohnologue sisters have different propensities to act as drivers of oncogenesis. For example, whereas most cases of cutaneous melanoma are driven by constitutively active *BRAF^V600E^* or *NRAS^Q61K^*, activated *KRAS* mutants are more common in pancreatic and colorectal cancers [19–22].

In a previous study, we investigated how mutations in cancer genomes are globally distributed across all ohnologue gene families. Strikingly, a few hundred ohnologue gene families were identified in which one gene is more frequently mutated at multiple positions compared with its ohnologue sisters in at least one cancer type. Moreover, in those ohnologue gene families that displayed such a ‘mutation-load skew’ in multiple cancers, the same gene carried the heaviest mutation load in each cancer, and usually the second-ranked gene was also the same in each cancer. These patterns indicate that specific ohnologues may be more important than their sisters as downstream non-mutated effectors in oncogenic signalling circuits in these cancers [23,24].

The group II PAK protein kinases, PAK4, PAK5 and PAK6, comprise one such mutation-load-skewed ohnologue family [24]. PAK4, PAK5 and PAK6 are effectors of Rho family small GTPases, and they phosphorylate regulators of cytoskeleton organisation, cell survival, cell cycle progression and motility [25–28]. In the genomes of specific cancers, namely breast, head-and-neck, liver, lung adenocarcinoma, squamous cell lung cancer, and melanomas, *PAK5* frequently carries non-synonymous point mutations, and small insertions and deletions; *PAK6* and *PAK4* have fewer; and *PAK4* is often amplified. Moreover, *PAK4* amplification is associated with poor prognosis in Ras-driven tumour models [23,24,27,28].

These genetic data are consistent with *PAK4* being more functionally relevant than *PAK5* and *PAK6* for these cancers [27,29], even though the three enzymes have similar domain architectures and intrinsic substrate specificities [17,30–33]. To investigate this hypothesis, here we compare the cellular regulation of PAK4, PAK5 and PAK6, and investigate their relative roles in melanoma cells.

In common with many ohnologues, PAK4, PAK5 and PAK6 can be phosphorylated and consequently interact with the phosphoprotein-binding 14-3-3 proteins. The 14-3-3s are dimeric and dock onto specific pairs of phosphorylated serine and threonine residues, which are usually in tandem within their target proteins [17,31]. Here, we identified further ohnologue protein kinases as 14-3-3-binding proteins. And, as a starting point to study their regulation, we identified the cellular stimuli, protein kinases, and phosphosites responsible for docking of PAK4, PAK5, and PAK6 onto 14-3-3 proteins.

## Results

### Human protein kinases are enriched in 14-3-3 binding proteins

**Figure 1.**
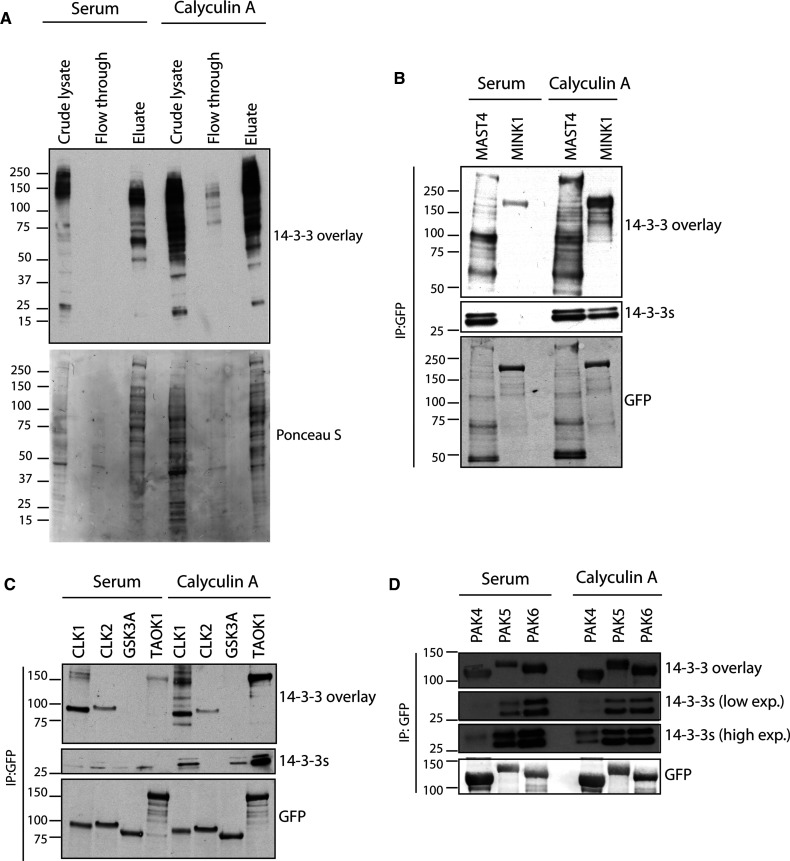
Protein kinases that also bind to 14-3-3 proteins. (**A**) Lysates of HEK293 cells, stimulated with serum and calyculin A, were clarified by protein-G Sepharose beads and incubated with gamma-phosphate-linked-ATP Sepharose in the presence of Mg^2+^. Proteins in cell lysates (30 µg), the flow through at the beginning of a salt wash (30 µg) and the elution pools from ATP-Sepharose beads were tested for their ability to bind to digoxygenin-labelled 14-3-3 in Far-Western overlay assays. Membranes were stained with Ponceau S to visualise proteins. Also, GFP-tagged protein kinases (**B**) MINK1, MAST4 (**C**), CLK1, CLK2, GSK3A, TAOK1 (**D**), PAK4, PAK5 and PAK6 were immunoprecipitated with GFP-Trap® beads from lysates (7.5 mg) of transfected HEK293 cells that had been serum-starved and calyculin A-treated, respectively, as indicated. Immunoprecipitated proteins were analysed for their ability to bind to digoxygenin-labelled 14-3-3 in Far-Western overlay assays, and for co-immunoprecipitation of endogenous 14-3-3s using the pan-14-3-3 antibody (K19, Santa Cruz). Anti-GFP signals indicate how much kinase was in each immunoprecipitate.

We investigated whether 14-3-3 binding to protein kinase ohnologues might be a more general phenomenon than previously realised, which would be in line with bioinformatic predictions [17,34]. We employed ATP-Sepharose, in which ATP is linked to the matrix via its gamma phosphate, to isolate active protein kinases and other ATP binding proteins from lysates of HEK293 cells, which had been stimulated with serum and the phosphatase inhibitor calyculin A [35]. After washing the columns, ATP-binding proteins were eluted in sample buffer and analysed by 14-3-3 Far-Western overlays, which showed that the elution pools containing eukaryotic protein kinases were enriched in 14-3-3 binding proteins (Figure 1A). The protein profiles of the ATP-Sepharose column eluate were distinct from that of the cell lysates, indicative of protein selection by the column (Figure 1A).

When queried with 14-3-3-Pred, kinases including CLK1, CLK2, TAOK1, MINK1, MAST4, GSK3A and group II PAK kinases scored most highly in terms of containing predicted 14-3-3-binding phosphosites [34]. Experimentally, CLK1, CLK2, TAOK1, MINK1 and MAST4, bound to 14-3-3s when these kinases were immunoprecipitated from lysates of transfected cells, as assessed by their co-immunoprecipitation with endogenous 14-3-3s and by direct binding to 14-3-3 in Far-Western overlays (Figure 1B,C). However, in contrast with previous findings of an interaction between GSK3B and 14-3-3 [36], we failed to detect any interaction between GSK3A and 14-3-3. As expected, the group II PAK kinases PAK4, PAK5 and PAK6 also bind to 14-3-3s when extracted from cells stimulated with either serum or calyculin A (Figure 1D).

### 14-3-3 binding and localisation of PAK4 to the leading edge of cells is mediated primarily via PKCα/β

**Figure 2.**
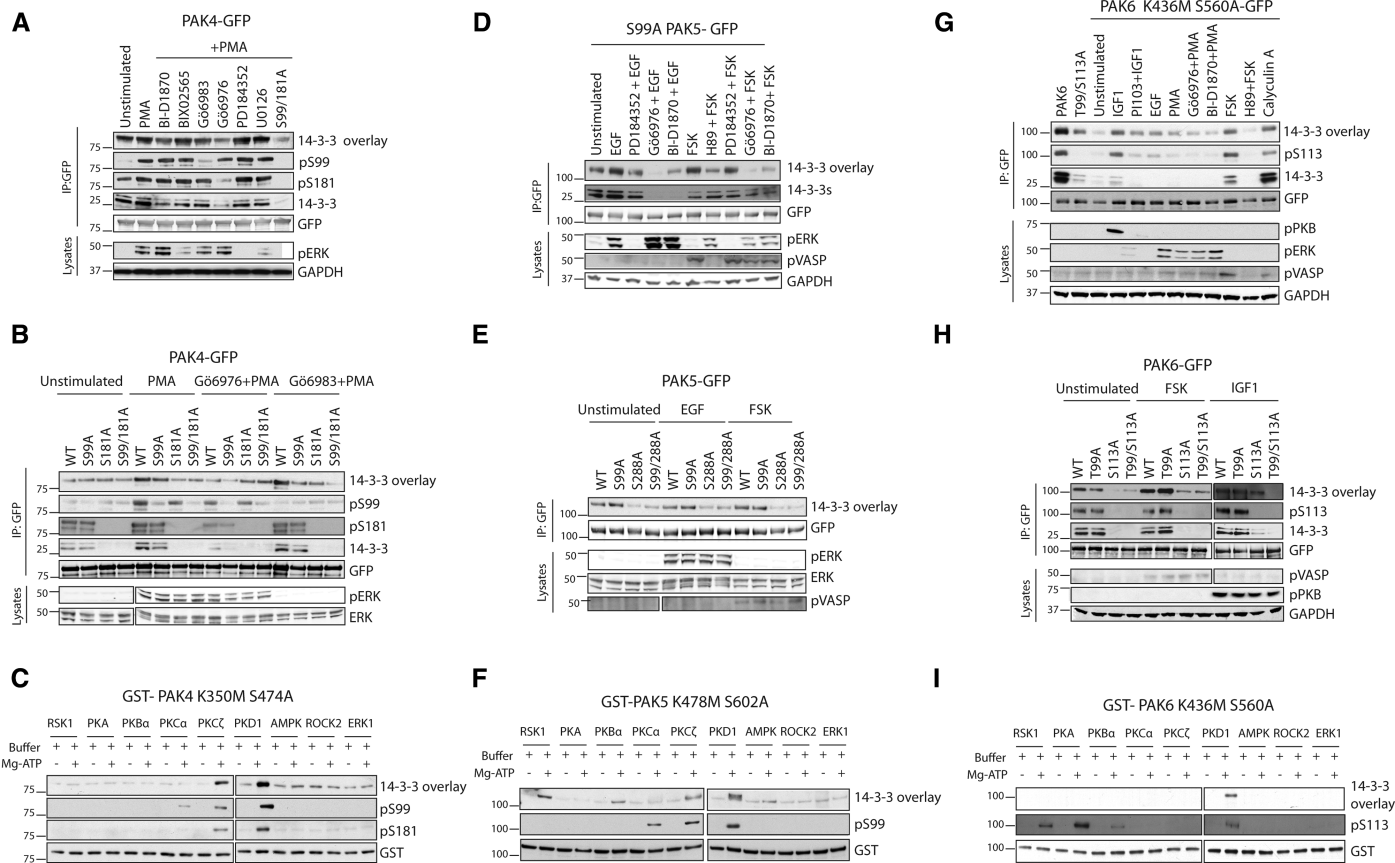
Regulation of binding of 14-3-3 to group II PAK kinases. (**A**) PAK4-GFP, wild-type and S99A/S181A mutant as indicated, was immunoprecipitated from lysates of stably transfected and induced HEK293 cells that had been stimulated with PMA in the presence and absence of compounds to inhibit p90RSK (BI-D1870 and BIX02565), PKCs (Gö6976 and Gö6983) and the Erk pathway (PD184352 and U0126) as indicated. The isolated proteins were analysed for binding to 14-3-3 proteins in a Far-Western overlay assay and for co-immunoprecipitation with endogenous 14-3-3s (K19 antibody, Santa Cruz). The phosphorylation status of Ser99 and Ser181 of PAK4 were assessed with phosphospecific antibodies. The amounts of PAK4 in immunoprecipitates were indicated using an anti-GFP antibody. Cell lysates (30 µg) were immunoblotted to assess the phosphorylation status of Erk1/2 (pThr202/Tyr204) and amount of GAPDH. (**B**) PAK4-GFP, wild-type and S99A/S181A mutant as indicated, was immunoprecipitated from lysates of transfected HEK293 cells that had been stimulated with PMA in the presence or absence of PKC inhibitors (Gö6976 and Gö6983). Cell lysates (30 µg) were immunoblotted to assess the phosphorylation status of Erk1/2 (pThr202/Tyr204) and total Erk1/2. Bars indicate the ends of gels for samples run on smaller gels than the others. (**C**) Bacterially expressed GST-PAK4 kinase-dead (K350M) mutant was phosphorylated *in vitro* using purified kinases (1 U/µg) as indicated with or without Mg-ATP. The ability of the protein to bind to 14-3-3s was assessed by Far-Western overlay assay, and phosphorylation of Ser99 with a phosphospecific antibody. (**D**) GFP-tagged PAK5-Ser99Ala was immunoprecipitated from stably transfected and induced cells that had been subject to the indicated stimuli and inhibitor combinations and analysed for binding to 14-3-3s. The levels of Erk-pThr202/Tyr204, total ERK and pSer157 VASP were assessed in cell lysates (30 µg). (**E**) PAK5-GFP, wild-type and mutants as indicated, immunoprecipitated from unstimulated, EGF-stimulated and FSK-stimulated cells were analysed for binding to 14-3-3s. Cell lysates were blotted for pERK, total ERK and pVASP. The levels of Erk-pThr202/Tyr204, total ERK and pSer157 VASP were assessed in cell lysates (30 µg). Bars indicate the ends of gels for samples run on smaller gels than the others. (**F**) Bacterially purified GST-PAK5 kinase-dead (K478M) mutant was phosphorylated *in vitro*with the indicated kinases, with or without Mg-ATP, and analysed for its ability to bind to 14-3-3s. The phosphorylation status of Ser99 was assessed with a phosphospecific antibody. (**G**) PAK6-GFP, wild-type and kinase-dead (K436M/S560A) mutant, was immunoprecipitated from stably transfected and induced cells that had been exposed to the indicated stimuli and inhibitor combinations and analysed for binding to 14-3-3s. Cell lysates (30 µg) were blotted for pSer473 PKB/Akt, pThr202/Tyr204 Erk and pSer157 VASP. (**H**) HEK293 cells expressing PAK6-GFP (wild-type and indicated mutants) were deprived of serum and stimulated with the adenylyl cyclase activator FSK and IGF1, and the isolated PAK6-GFP tested for binding to 14-3-3. The phosphorylation status of Ser113 was monitored with a phosphospecific pSer113-PAK6 antibody. Bars indicate the ends of gels for samples run on smaller gels than the others. (**I**) GST-tagged PAK6 kinase-dead mutant was phosphorylated *in vitro* with purified kinases, with or without Mg-ATP, and the consequent ability to bind to 14-3-3 and phosphorylation status were analysed. Data quantification is in Supplementary Figure S2.

Consistent with previous findings [17,31], 14-3-3 binds to PAK4-GFP in stable transfectants and to endogenous PAK4 when cells are stimulated by phorbol 12-myristate 13-acetate (PMA), as determined by co-immunoprecipitation of PAK4 with endogenous 14-3-3s and by the ability of exogenous 14-3-3 to bind directly to the immunoprecipitated PAK4 in far-Western assays (Figure 2A and Supplementary Figures S2A, S3A).

PMA activates cellular PKC (conventional and novel)–PKD and MEK–Erk–p90RSK signalling. The PMA-stimulated binding of 14-3-3 to PAK4 was inhibited when cells were pre-incubated with Gö6976, a selective inhibitor of PKCα/β and PKD. In contrast, MEK inhibitors such as PD184352 and U0126, and the p90RSK inhibitors BI-D1870 and BIX02565 had little or no effect on 14-3-3 binding to PAK4 (Figure 2A and Supplementary Figure S2A).

Antibodies were raised to recognise Ser99 and Ser181 of PAK4 only when these residues are phosphorylated. Use of these phospho-specific antibodies revealed that PMA stimulated the phosphorylation of Ser99 and to a lesser extent Ser181 (Figure 2A,B). A pan-PKC inhibitor Gö6983 abolished phosphorylation of Ser99 but had no effect on Ser181. In contrast, the PKCα/β and PKD inhibitor Gö6976 prevented PMA-stimulated phosphorylation of Ser181 of PAK4 (Figure 2A,B and Supplementary Figure S2A). Mutation analysis indicated that phosphoSer181 was the dominant 14-3-3-binding site (Figure 2B and Supplementary Figure S2A), consistent with the inhibitory effect of Gö6976 on the interaction between 14-3-3 and PAK4 (Figure 2B and Supplementary Figure S2A).

Basal binding of 14-3-3 to PAK4 in unstimulated mammalian cells due to trace phosphorylation of Ser181 was observed, as was low level phosphorylation of Ser181 in bacterially expressed PAK4. These basal phosphorylations of Ser181 were attributable to autophosphorylation and were eliminated when experiments were repeated with a kinase-dead mutant (K350M–S474A) of PAK4. However, when transfected cells were stimulated with PMA, the phosphorylations of Ser99 and Ser181 were comparable for both wild-type PAK4 and K350M–S474A–PAK4 (Supplementary Figure S3A).

When bacterially expressed K350M–S474A–PAK4 was phosphorylated with candidate protein kinases, PKCζ and PKD1 phosphorylated Ser99 and Ser181 of PAK4, making it capable of binding to 14-3-3 (Figure 2C and Supplementary Figure S2D). Together with the effects of Gö6983 and Gö6976 (Figure 2A and Supplementary Figure S2A), these results suggest that a different PKC and PKD phosphorylate Ser99 and Ser181 of PAK4, respectively, in PMA-stimulated cells.

### PAK5 that is phosphorylated by p90RSK consequently binds to 14-3-3

The binding of 14-3-3 to PAK5 was also stimulated by PMA. In contrast with PAK4 however, the interaction between PAK5 and 14-3-3 was inhibited by pre-treatment with the non-specific PKCα/β and PKD inhibitor Gö6976, and with the selective p90RSK inhibitor BI-D1870 (Supplementary Figure S4A). Ser99 of PAK5, which aligns with Ser99 of PAK4, was phosphorylated in PMA-stimulated cells, and this was prevented by Gö6983 (Supplementary Figure S4A). Thus, the regulation of phosphoSer99 is conserved in PAK4 and PAK5. However, mutation of Ser99 to alanine had a negligible effect on PMA-stimulated 14-3-3 binding to PAK5 (Supplementary Figure S4B).

To identify the second 14-3-3 binding site of PAK5, candidate sites that conform to 14-3-3-binding consensus motifs were mutated to alanine. Mutation of Ser288 to alanine significantly decreased the binding of 14-3-3 to PAK5-Ser99Ala that occurred in response to serum, epidermal growth factor (EGF), PMA and adenylate cyclase activator FSK, while mutation of Ser150, Ser226, Ser286 and Ser290 had no effect (Figure 2D,E and Supplementary Figures S2B, S4B). The EGF-induced binding of 14-3-3 to PAK5-Ser99Ala was inhibited by pre-treatment with Gö6976 and BI-D1870, whereas Gö6976, BI-D1870 and H-89 inhibited its FSK-stimulated binding to 14-3-3 (Figure 2D). These results suggest that phosphorylation of Ser288 of PAK5 occurs in response to EGF through PKC/PKD or p90RSK.

Ser99 of PAK5 was phosphorylated by PKCα, PKCζ and PKD *in vitro* (Supplementary Figure S2E). In contrast, phosphorylation by p90RSK1, PKBα, PKCζ and PKD1 rendered PAK5 capable of binding to 14-3-3 (Figure 2F). LC–MS/MS analyses of tryptic digests of the *in vitro*-phosphorylated PAK5 identified several phosphorylated sites of which peptides containing phosphoSer286/288/290 were enriched when PAK5 kinase-dead mutant was phosphorylated with p90RSK1, PKBα and PKD1 (Supplementary Table S1). The MS analysis could not differentiate between phosphorylation of Ser286, Ser288 or Ser290. Although FSK did induce binding of 14-3-3 to PAK5-Ser99Ala in cells (Figure 2D), phosphorylation by PKA did not mediate high 14-3-3 binding to PAK5 *in vitro* (Figure 2F). Together, these data suggest that phosphoSer288 is the high affinity 14-3-3 binding phosphosite of PAK5. In contrast, phosphorylation of Ser99 of PAK5 correlates with, but makes little or no contribution to, its binding to 14-3-3 (Figure 2E and Supplementary Figure S2B).

### PAK6 binds to 14-3-3 when phosphorylated in response to IGF1 and cyclic-AMP elevating agents

The binding of 14-3-3 to a kinase-dead K436M S560A mutant of PAK6 was stimulated by insulin-like growth factor 1 (IGF1), which activates PI 3-kinase–PKB/Akt and by the adenylate cyclase activator FSK. Binding of 14-3-3 to PAK6 paralleled its phosphorylation on Ser113. PI-103, the PI 3-kinase and mTOR inhibitor, markedly decreased the phosphorylation of Ser113 and binding of 14-3-3 to PAK6 in response to IGF1, and H-89 a non-specific but potent inhibitor of PKA blocked its Ser113 phosphorylation and binding to 14-3-3 in response to FSK (Figure 2G). IGF1- and FSK-induced binding of 14-3-3 to PAK6 was inhibited by mutation of Ser113 to alanine (Figure 2H and Supplementary Figure S2C). Consistent with these results, PKA and PKBα phosphorylated Ser113 *in vitro* (Figure 2I and Supplementary Figure S2E). These data suggest that IGF1 via PKB, and FSK via PKA, stimulate phosphorylation of Ser113 of PAK6 and its consequent binding to 14-3-3.

The residue corresponding to Ser99 in PAK4 and PAK5 is Thr99 in PAK6. Thr99 of PAK6 has been reported to be phosphorylated in a large-scale proteomic study [37], and substitution of Thr99 with alanine slightly decreased 14-3-3 binding of PAK6 (Figure 2H). However, we failed to identify peptides containing phosphoThr99 by mass spectrometric analysis of digests of PAK6 isolated from serum-, IGF1- and FSK-stimulated cells. Moreover, the phosphospecific antibody for phosphoSer99 of PAK4 was not useful for monitoring the phosphorylation of Thr99 of PAK6. Again, these data are consistent with PKA- and PKB/Akt-phosphorylated Ser113 being the predominant 14-3-3 binding phosphosite of PAK6.

In summary, the binding of 14-3-3 to PAK4, PAK5 and PAK6 is differentially regulated by multiple regulatory inputs (Supplementary Figure S5).

### PAK4, PAK5 and PAK6 are translocated between different subcellular locations when their 14-3-3-binding sites are phosphorylated

**Figure 3.**
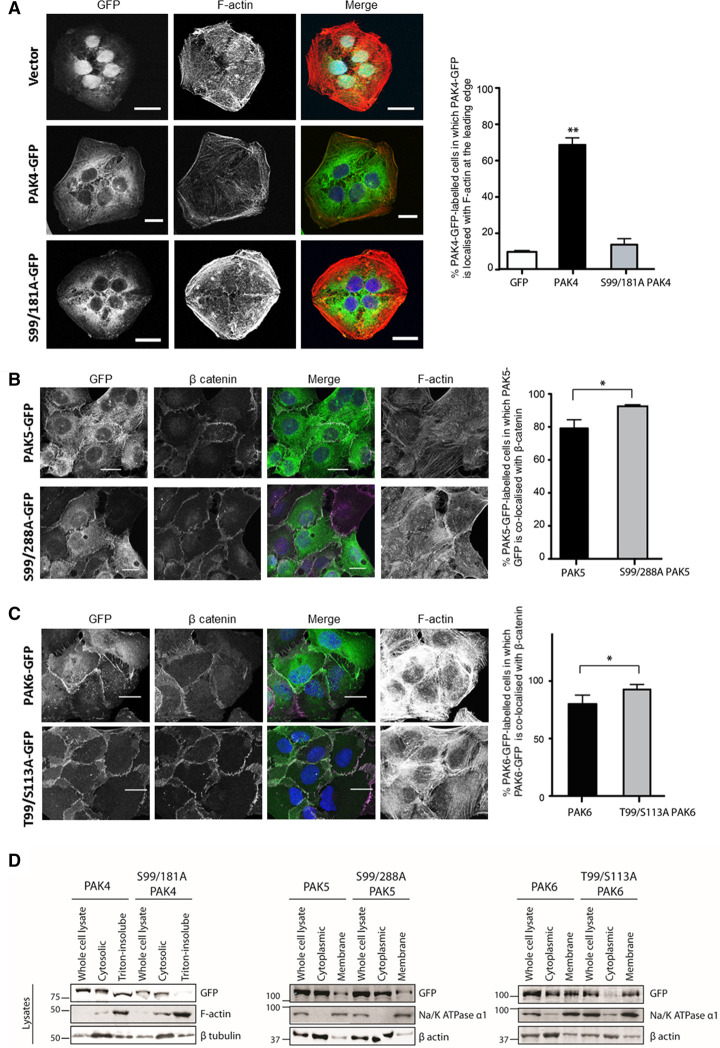
Localisation of PAK4, PAK5 and PAK6. U2OS FlpIn TRex cells were stably transfected and induced to expression of GFP (vector only), PAK4 (**A**), PAK5 (**B**) and PAK6 (**C**) wild-type and mutant for 14-3-3 binding sites were induced in U2OS FlpIn TRex cells using 0.2 µg/ml tetracycline for 16 h. Cells were fixed in 4% paraformaldehyde for 20 min, permeabilised and stained for actin using Alexa Fluor 594 phalloidin and nucleus using DAPI. Coverslips were mounted onto slides and images were taken using Zeiss LSM710 confocal microscopy, scale bars 10 µm (**A**) and 20 µm (**B** and **C**). Numbers of GFP expressing cells were counted and the localisation was quantitated (*n* ≥ 60 × 3 experiments, indicating standard deviations) by indicating whether signal was observed with F-actin at the leading edge (**A**) or co-localised with β-catenin at cell junctions (**B** and **C**). The localisations were scored blind to the source of the signals. The bar graphs were plotted using GraphPad prism 5.0, * *P* < 0.05 and ** *P* < 0.001 (ANOVA, student *t*-test). (**D**) U2OS FlpIn TRex cells stably expressing the indicated GFP-tagged wild-type or mutant kinases were fractionated as described in the Materials and Methods, and cell lysates were subjected to Western blotting with the indicated antibodies.

Wild-type PAK4 localises to both the cytoplasm and leading edge of the cell, while PAK4 with mutated 14-3-3 binding sites (S99A/S181A) is retained in the cytoplasm (Figure 3A), as is the S99A mutant of PAK4 [31,38]. Furthermore, wild-type PAK4 was extracted in cytosolic and Triton-X100-insoluble cytoskeletal fractions of cell lysates, whereas the S99A/S181A mutant was exclusively in the cytosolic fraction (Figure 3D). The kinase-inactive K350M/S474A mutant of PAK4 localised predominantly to the cytoplasm and to the perinuclear region (Supplementary Figure S6).

Wild-type PAK5 localises to the cytoplasm and cell–cell junctions, and the cell–cell junction localisation was enhanced for its S99A/S288A mutant that does not bind to 14-3-3. Consistent with previous studies [39,40], the cytoplasmic PAK5 was associated with punctate structures (Figure 3B).

Wild-type PAK6 is concentrated at cell–cell junctions and in the cytoplasm [41], whereas PAK6 with mutated 14-3-3 binding sites localises exclusively at cell–cell junctions. Moreover, wild-type PAK6 was found both in cytoplasmic and membrane fractions, while the PAK6-T99A/S113A mutant was found exclusively in the membrane fraction of cell lysates (Figure 3D). The kinase-inactive mutants of PAK5 and PAK6 also localised to the cell–cell junctions, which suggests that their kinase activities do not determine their cell–cell junction localisation (Supplementary Figure S6).

In summary, these data indicate that phosphorylation and binding to 14-3-3 causes PAK4 to move to the leading edge of moving cells, affects the cell–cell junction location of PAK5, and promotes the release of PAK6 from membrane into cytoplasm.

### Group II PAK kinases have different roles in melanoma cells

**Figure 4.**
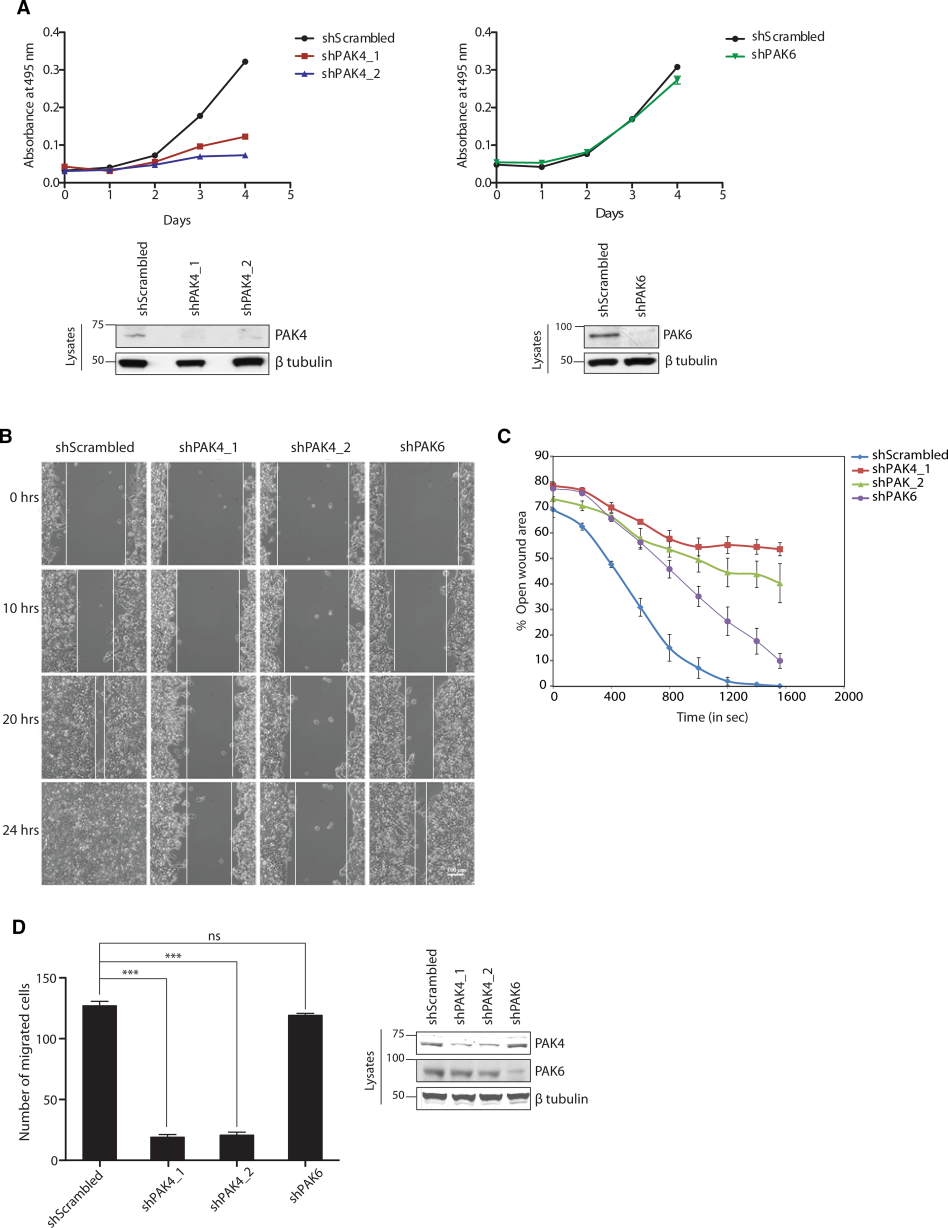
PAK4 knockdown impairs cell proliferation and migration of melanoma cells. (**A**) SKMEL13 cells (2000 cells/well) with PAK4 and PAK6 knockdown were seeded onto 96-well plates and allowed to attach overnight. The cell proliferation assay was carried out over a period of 4 days in triplicates using the CellTiter96® Aqueous Non-Radioactive Cell Proliferation Assay kit (Promega) as described in the Materials and Methods section. The efficiency of PAK4 and PAK6 knockdown was analysed by Western blotting. (**B**) PAK4- and PAK6-knockdown SKMEL13 cells were seeded into the chambers of the IBIDI Self-Insertion Inserts. Cells were allowed to attach onto the plate overnight. The inserts were then removed and a wound-healing assay was carried out in triplicates. Snapshots taken at different time points (*t* = 0, 10, 20 and 24 h) by time-lapse microscopy were used as representative images for comparison between the migration properties of cells knockdown for PAK4 and PAK6 expression and cells treated with scrambled shRNA as a control, scale bar = 100 µm. *Right*: The graph represents percentage wound area closure at different time points. Percentage wound closure was calculated using the software Tscratch. Error bars represent standard deviation (*n* = 6). (**C**) SBcl2 melanoma cells silenced for PAK4 and PAK6 expression (3 × 10^5^ cells/ml) in serum free medium were seeded on the top of the transwell chamber and medium containing 10% serum was loaded onto the bottom of the chamber. After 20 h, unmigrated cells on top of the chamber were scraped off and the cells at the bottom of the transwell were fixed and stained using diff quick stain kit (Gentaur). Images were taken at 10× magnification, migrated cells were counted and the graph was plotted. Data are represented as mean ± SEM, *** *P* < 0.0001, and not significant (ns), ANOVA followed by Tukey's test for pairwise comparison. *Right*: Western blot represents the level of PAK4 and PAK6 knockdown, with β-tubulin as the loading control. (**D**) Effects of silencing PAK4 and PAK6 in SBcl2 cells on migration (left), and evidence of knock-down of these kinases (right). Bars indicate the number of cells migrating through Matrigel relative to control cells treated with scrambled shRNA.

PAK4 and PAK6 are expressed to different levels in different melanoma cell lines, while PAK5 expression is low or undetectable in these cells (Supplementary Figure S10). Silencing of PAK4 expression in melanoma cells impaired proliferation or survival consistent with previous findings in other cell types [42,43], whereas silencing PAK6 had little or no effect (Figure 4A and Supplementary Figure S8). Group II PAK kinases have well studied roles in cell migration [25,44]. We therefore analysed the effect of silencing PAK4 and PAK6 expression on migration of melanoma cells. Silencing PAK4 in *BRAF^V600E^* SKMEL13 cells impaired cell migration in 2D wound healing assays, consistent with previous findings in other cell types [43,45], while knocking down PAK6 expression had little effect (Figure 4B). SBcl2 melanoma cells carrying the *NRAS^Q61K^* mutation are invasive, and the effects of knocking down PAK4 and PAK6 on their migration was analysed using a Transwell Matrigel migration assay. Silencing PAK4 in SBcl2 cells had a profound effect on migration, with only 15% of the cells migrating through Matrigel relative to control cells treated with scrambled shRNA. In contrast, knocking down PAK6 expression had little effect on migration of SBcl2 cells (Figure 4C,D). These results suggest that PAK4, and not PAK6, has a predominant role in regulating cell migration in SKMEL13 and SBcl2 melanoma cell lines.

**Figure 5.**
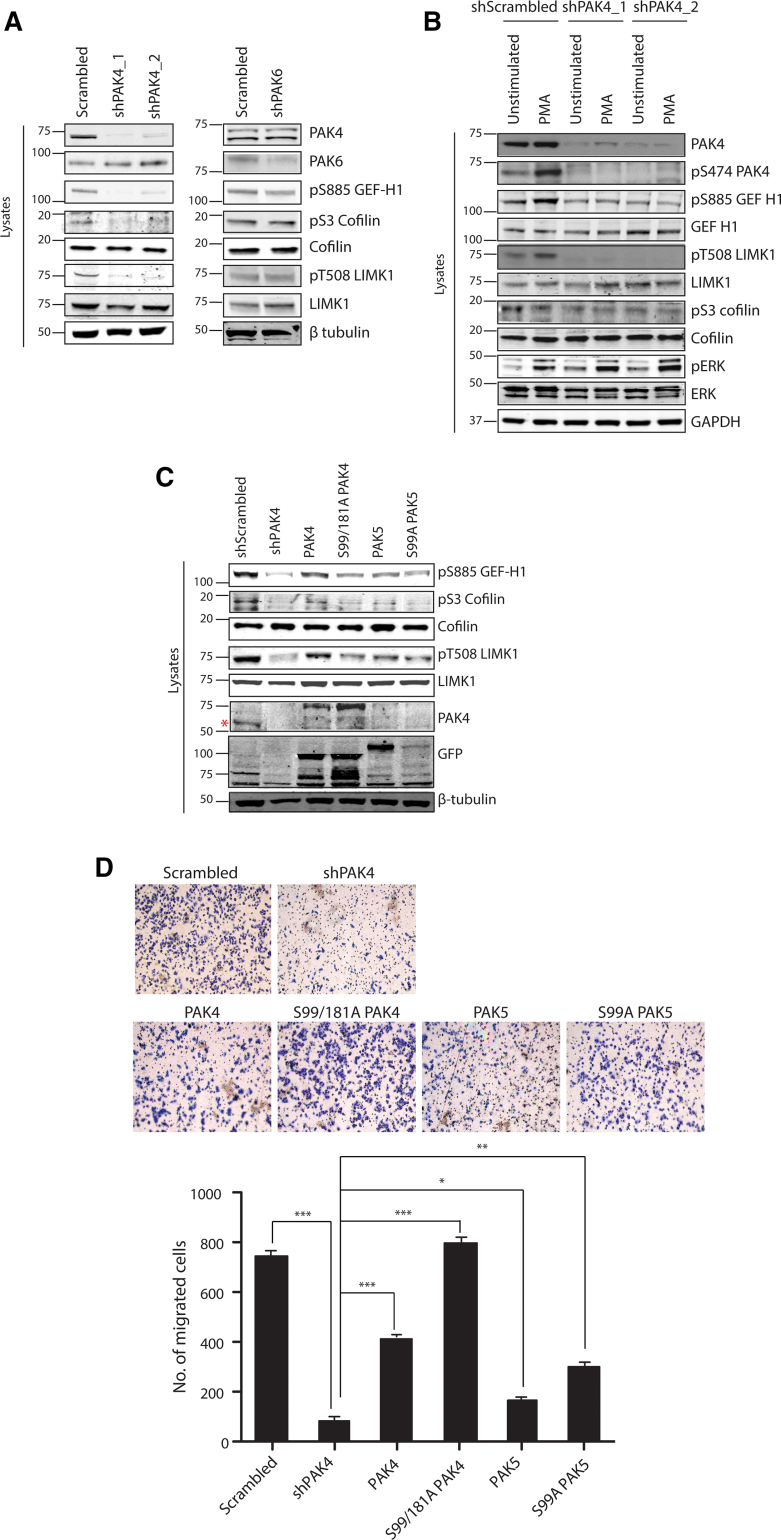
Role of PAK4, PAK5 and PAK6 in melanoma cells. (**A**) The phosphorylation status of GEF-H1, LIMK1 and cofilin in lysates of SKMEL1 cells in which PAK4 and PAK6 had been stably knocked down was analysed using pSer885-GEF-H1, pThr508-LIMK1 and pSer3-cofilin antibodies, with β-tubulin as the loading control. (**B**) SKMEL13 cells treated with scrambled shRNA were serum starved and stimulated with PMA with and without pre-incubation with Gö6976 and PAK4 inhibitor, PF-3758309. (**C**) PAK4 knockdown was rescued by overexpressing PAK4 and PAK5 wild-type and mutant for 14-3-3 binding sites. The phosphorylation status of downstream targets was analysed by blotting cell lysates (30 µg) for pSer885-GEF-H1, pThr508-LIMK1 and pSer3-cofilin, with β-tubulin as loading control. Expression levels of GFP-tagged PAK4 and PAK5 wild-type and mutants were analysed by using anti-GFP antibody. (**D**) SBcl2 melanoma cells were treated with scrambled and PAK4 (targeting 3′UTR) shRNA. PAK4 knockdown cells were transfected to express PAK4 and PAK5, wild-type and mutant proteins with each pair of 14-3-3 binding sites substituted by alanine. Forty eight hours post-transfection, cells (3 × 10^5^ cells/ml) suspended in serum free medium were seeded on the top of transwell chambers and medium containing 10% serum was loaded onto the bottom of the chamber. After 20 h, unmigrated cells on top of the chamber were scraped off and the cells at the bottom of the transwell were fixed and stained using diff quick stain kit (Gentaur). Assays were carried out in duplicate and images were taken at 10× magnification in at least three fields. Representative images are shown. Migrated cells were counted and data are represented as mean ± SEM; * *P* < 0.05, ** *P* < 0.001 and *** *P* < 0.0001; ANOVA followed by Tukey's test for pairwise comparison.

We next investigated the phosphorylation status of protein substrates of PAK4 such as LIMK1, Cofilin and GEF H1 that are implicated in cell migration. Consistent with the effects of PAK4 on cell migration, silencing PAK4 decreased the basal and PMA-stimulated phosphorylation of LIMK1 at Thr508, thereby inhibiting phosphorylation by LIMK1 of Ser3 of cofilin in SKMEL13 cells. In contrast, phosphorylation of LIMK1 and cofilin was unaffected by PAK6 knockdown in these cells (Figure 5A). Furthermore, silencing PAK4 expression also significantly decreased the phosphorylation of GEF H1 at Ser885 (Figure 4A). Ser885 phosphorylation inhibits the activity of GEF H1 and thereby decreases levels of active GTP-loaded RhoA in cells [46,47]. These findings are consistent with the increased formation of actin stress fibres, and of focal adhesions, as evidenced by enhanced paxillin staining, which were observed in cells lacking PAK4, compared with controls treated with scrambled shRNA (data not shown).

Interestingly, stimulation with PMA increased phosphorylation of GEF-H1 and LIMK1, while this was inhibited by the PAK4 inhibitor PF-3758309 and the PKC/PKD inhibitor Gö6976 (Supplementary Figure S9). Consistent with these results, the knockdown of PAK4 inhibited the PMA-induced phosphorylation of GEF H1 and LIMK1 (Figure 5B), which was rescued by expression of PAK4 but to a lesser extent by expression of PAK4 that cannot bind to 14-3-3s (Figure 5C). In addition to the regulation of 14-3-3 binding sites, stimulation with PMA was found to enhance phosphorylation of the activation loop serine residue (S474) of PAK4 (Figure 5B). This finding suggests that PKC signalling regulates cell migration by altering phosphorylation, 14-3-3 binding and localisation of PAK4, and by enhancing its kinase activity.

We next investigated whether the other cellular effects of knocking down PAK4 could be rescued by ectopic expression of type II PAKs. PAK4 expression partially rescued the effect of loss of endogenous PAK4 on cell migration in SBcl2 melanoma cells (Figure 5D). Although the kinase activities of PAK4 wild-type and 14-3-3 binding mutant were similar, the S99/181A mutant rescued the effect of PAK4 knockdown on cell migration to a greater extent than wild-type (Figure 5D). PAK5 was not detected in melanoma cell lines tested, and silencing PAK5 expression in these cell lines had no effect (Supplementary Figure S10). Moreover, overexpressing PAK5 wild-type and Ser99Ala mutant in SBcl2 cells caused only partial reversion of the phenotype of PAK4 knockdown although the Ser99Ala mutant was more effective (Figure 5D).

Taken together, these results suggest that PAK4 has a predominant role in regulating cytoskeletal organisation and migration in melanoma cells. They also indicate that while PAK4 that cannot bind to 14-3-3 has a lesser effect than wild-type PAK4 in mediating the PMA-stimulated phosphorylation of downstream substrates, it has a greater effect in maintaining cell migration.

## Discussion

In addition to PAK4, PAK5 and PAK6, 5 out of 6 protein kinase ohnologues tested here (CLK1, CLK2, TAOK1, MINK1 and MAST4), display phosphorylation-dependent interactions with 14-3-3. These findings indicate that many protein kinases are ohnologues that bind to 14-3-3s, and understanding the 14-3-3 regulation of these ohnologue protein families may help interpret the logic of how ohnologue signalling networks are rewired by genomic disruptions in cancers [48,49]. Here, we identify PAK4 as an essential effector of an oncogenic signalling network in melanoma cells, consistent with emerging data pinpointing this specific group II PAK as an effector in Ras-driven tumours [27,28].

PAK4 is phosphorylated on Ser99 in response to phorbol ester, which activates conventional PKCs (α, β, γ), novel PKCs (δ, ε, η, θ) and PKDs (1, 2, 3) [50,51]. Phorbol ester-stimulated phosphorylation of Ser99-PAK4 phosphorylation is blocked by the pan-PKC inhibitor Gö6976, but not the PKCα/β and PKD inhibitor Gö6983 (δ, ε, η, θ). Therefore, Ser99 is phosphorylated by PKC γ, δ, ε, η and/or θ. Conversely, the phorbol ester-stimulated phosphorylation of Ser181-PAK4 is inhibited by Gö6983, but not Gö6976, indicating its phosphorylation by a PKD. The doubly phosphorylated PAK4 consequently docks onto a 14-3-3 dimer, with phosphoSer181 having higher binding affinity than phosphoSer99. These regulations cause PAK4 to be translocated to the leading edge of migrating cells, where it phosphorylates substrates including Ser885 of GEF H1, and Thr508 of LIMK1 that in turn inhibits its phosphorylation of Ser3 of cofilin, thereby regulating actin cytoskeletal dynamics [52–54].

In contrast with PAK4, PAK5 was undetectable in the melanoma cells and its ectopic expression did not rescue the loss of PAK4. PAK6 silencing had little or no effect on the tested melanoma cell phenotypes. While the three sisters, PAK4, PAK5 and PAK6, share one common 14-3-3-binding site (phosphoSer99 in PAK4), the second 14-3-3-binding site differs for each sister and is phosphorylated by different upstream protein kinases: phosphoSer181–PAK4 (RDKRPL(pS_181_)GPD) is phosphorylated by a Gö6976-inhibited kinase, most likely a PKD; phosphoSer288–PAK5 (MRQRSR(pS_288_)GSG) is phosphorylated in response to EGF by either PKC/PKD or p90RSK; whereas 14-3-3 docks onto phosphoSer113–PAK6 (SRRRAQ(pS_113_)LGL) that is phosphorylated by PKA and PKB/Akt. Furthermore, interactions of 14-3-3 with phosphoPAK4, phosphoPAK5 and phosphoPAK6 result in translocations of each kinase from and to different subcellular locations: PAK4 from the cytoplasm to the leading edge of cells, PAK5 from cell–cell junctions to cytoplasmic puncta, and PAK6 from cell–cell junctions to a diffuse cytoplasmic distribution.

Ser99 and Ser181 of endogenous PAK4 are also phosphorylated in SKMEL13 (*BRAF^V600E^*) and SBcl2 (*NRAS^Q61K^*) melanoma cells, indicating that relevant PKC(s) and PKD(s) are active in these cells. Moreover, silencing of PAK4 impairs viability, migration, and invasive behaviour of melanoma cells. These phenotypes were rescued by ectopic expression of PAK4, and to a greater extent by a 14-3-3 binding-deficient form of PAK4. However, further work will be required to understand why the 14-3-3-binding deficient form of PAK4 is more effective than wild-type PAK4 in supporting the melanoma cell phenotype, particularly given that roles have been identified for PKCs, including PKCδ, in survival and metastatic invasion of melanoma cells carrying *RAS* and *BRAF* driver mutations [12,55–62].

Together these findings indicate that only PAK4, but not PAK5 and PAK6, can complement the requirement for survival and migration of melanoma cells. Thus, it makes sense that the *PAK4* gene should be selected for preservation and amplification in cancers whose oncogenic signalling networks require this enzyme as a downstream effector.

## Materials and methods

### Reagents

Digoxygenin-O-methylcarbonyl-ε-aminocaproic acid N-hydroxysuccinimide (DIG) ester (#11333054001), anti-DIG-POD Fab fragments (#11207733910) and cOmplete Mini EDTA-free protease inhibitor tablets (#1697498) were from Roche; Coomassie Protein Assay reagent from Thermo Scientific, polyethylenimine (PEI) from Polysciences Inc; Protein G-Sepharose, glutathione-Sepharose and enhanced chemiluminescence Western blotting kit (RPN2106) were from Amersham Bioscience; Precast NuPAGE® polyacrylamide Bis-Tris gels, Lipofectamine® 2000 transfection reagent, hygromycin, blasticidin, zeocin, puromycin, 4′,6-diamidino-2-phenylindole dihydrochloride (DAPI) (D1306) and Alexa Fluor® Phalloidin (A12381) were from Invitrogen; sequencing-grade trypsin from Promega; and microcystin-LR was purchased from Professor Linda Lawton (Robert Gordon University, Aberdeen, U.K.).

### Antibodies

The antibodies that recognise phosphorylated sites on group II PAK kinases are available from https://mrcppureagents.dundee.ac.uk/ and were raised in sheep against the following synthetic phosphopeptides: CSVTRSNpSLRRDSP (residues 93 to 105 plus Cys for coupling, where pS represents pSer99 of PAK4 and PAK5; sheep S632D, first bleed); CRDKRPLpSGPDVGT (residues 175 to 187 plus Cys for coupling, where pS represents pSer181 of PAK4; sheep S637D, third bleed) and CSRRRAQpSLGLLGD (residues 107 to 119 plus Cys for coupling, where pS represents pSer113 of PAK6; sheep S638D, 3rd bleed). Anti-14-3-3 antibody was from Santa Cruz. Antibodies that recognise PAK4, LIMK1, cofilin, pSer473 on PKB/Akt, pSer80 on ACC, pThr202/Tyr204 on ERK1/2, pSer157on VASP, pThr508/505 on LIMK1/2, pSer885 on GEF-H1 and pSer3 on cofilin were from Cell Signalling Technology. Anti-PAK6 and anti-β-tubulin antibodies were from Abcam and anti-GFP-Dylight-800 from Rockland. Digoxygenin (DIG)-14-3-3 probes were prepared as described previously (Moorhead et al. 1999), used in far-Western overlay assays (which are similar to Western blots, but where DIG-14-3-3 takes the place of primary antibody) and detected using anti-DIG antibody conjugated with HRP (Roche). GFP-Trap® agarose beads were from Chromotek.

### Plasmids

Recombinant DNA procedures, restriction digests, ligations and PCR, were performed using standard protocols. All PCR reactions were carried out using KOD Hot Start DNA polymerase (Novagen). DNA sequencing was performed by the Sequencing Service, School of Life Sciences, University of Dundee (www.dnaseq.co.uk) using Applied Biosystems Big-Dye Ver 3.1 chemistry on an Applied Biosystems model 3730 automated capillary DNA sequencer.

Vectors for expression of proteins in mammalian cells were pcDNA5/FRT/TO (for stable integration of plasmid and tetracycline induced expression) modified to express C-terminal GFP-tagged forms of PAK4, PAK5 and PAK6. In-house numbers for the reference clones, available from https://mrcppureagents.dundee.ac.uk/, are as follows: PAK4-GFP (DU40959), PAK4 Ser99Ala-GFP (DU40970), PAK4 Ser181Ala-GFP (DU40992), PAK4 Ser99/181Ala-GFP (DU45219), PAK4 Ser474Ala-GFP (DU40971), PAK4 Ser162Ala-GFP (DU40991), PAK4 Lys350Met Ser474Ala-GFP (DU45854), PAK5-GFP (42406), PAK5-Ser99Ala-GFP (DU45453), PAK5 Ser150Ala-GFP (DU45454), PAK5 Ser226Ala-GFP (DU45679), PAK5 Ser99/Ser226-GFP (DU40970), PAK5 Ser288Ala-GFP (DU42453), PAK5 Ser99/Ser288Ala-GFP (DU45565), PAK5 Lys478Met Ser602Ala-GFP (DU42453), PAK6-GFP (DU42405), PAK6 Thr99Ala-GFP (DU45455), PAK6 Ser113Ala-GFP (DU45456), PAK6 Thr99/Ser113Ala-GFP (DU45560) and PAK6 Lys436Met Ser560Ala-GFP (DU45855). The clones PAK5 Ser286Ala-GFP (DCP1754), PAK5 Ser99/Ser286Ala-GFP (DCP1755) and PAK5 Ser288/290Ala-GFP (DCP1600) were from Dundee Cell Products, U.K.

The pGEX6P vector was used for expressing the following human proteins with N-terminal GST tags in BL21 *E. coli* induced with IPTG (isopropyl β-d-1-thiogalactopyranoside): GST-PAK4 Lys350Met Ser474Ala (DU45856), GST-PAK5 Lys478Met Ser602Ala (DU45863) and GST-PAK6 Lys436Met Ser506Ala (DU45862).

### Culture, treatment and lysis of HEK293 and melanoma cells

HEK293, U2OS and SBcl2 cells were grown in DMEM (Dulbecco's modified Eagle's medium), and SKMEL13 melanoma cells in RPMI-1640, supplemented with 10% (v/v) FBS (foetal bovine serum), 2 mM l-glutamine, 1% (v/v) penicillin and 1% (v/v) streptomycin.

Cells grown on 10 cm or 15 cm diameter dishes were transiently transfected with the plasmids indicated (PEI method). From 48 to 60 h post-transfection, cells were deprived of serum for 12 h (unstimulated), then stimulated with IGF1 (50 ng/ml for 15 min), EGF (50 ng/ml for 15 min), PMA (100 ng/ml for 30 min), FSK (20 µM for 30 min), A23187 (10 µM for 15 min), A769662 (50 µM for 60 min) and protein phosphatase inhibitor calyculin A (50 ng/ml for 15 min). Where indicated, prior to stimulations, cells were incubated with PI-103 (1 µM for 30 min), Gö6976 (1 µM for 30 min), Gö6983 (1 µM for 30 min), BI-D1870 (10 µM for 30 min), PD184352 (2 µM for 60 min), U0126 (10 µM for 60 min) and H-89 (30 µM for 30 min). The medium was aspirated; cells were washed in ice-cold PBS and lysed in 0.45 ml of ice-cold Triton X-100 lysis buffer comprising 50 mM Tris–HCl (pH 7.5 at 4°C), 0.12 M sodium chloride, 1% Triton X-100, 0.27 M sucrose, 1 mM EDTA (pH 8), 1 mM EGTA (pH 8), 50 mM sodium fluoride, 10 mM β-glycerophosphate, 5 mM sodium pyrophosphate, 1 mM sodium orthovanadate, 1 mM benzamidine, 1 mM phenylmethylsulfonyl fluoride (PMSF), 0.1% β-mercaptoethanol, 1 µM microcystin-LR, and one mini cOmplete^TM^ protease inhibitor tablet per 10 ml of lysis buffer. Lysates were clarified by centrifugation (16,000***g*** for 20 min at 4°C), snap frozen and stored at −80°C. Protein concentrations were determined using Coomassie Protein Assay reagent.

### Immunoprecipitations and GFP-Trap® pulldowns

Cell lysates were precleared by incubating with Protein-G agarose/Sepharose beads for 1 h at 4°C. For immunoprecipitating GFP-tagged proteins, precleared lysates (1 to 5 mg) were incubated with GFP-Trap-agarose beads (20 µl) for 2 h or overnight at 4°C, washed twice in low salt buffer (50 mM Tris–HCl pH 7.5, 0.15 M NaCl, protease inhibitors) and once in high salt buffer (50 mM Tris–HCl pH 7.5, 0.5 M NaCl, protease inhibitors). ATP-Sepharose pulldowns were performed by incubating clarified cell lysates (400 µg) with ATP-Sepharose beads, in which ATP is linked to the matrix via its gamma phosphate group, for 2 h at 4°C and washed thrice with low salt buffer (50 mM Tris–HCl pH 7.5, 0.15 M NaCl, protease inhibitors). Immunoprecipitated proteins were eluted in 2% LDS sample buffer with 2% mercaptoethanol.

### Generation of stably transfected U2OS Flp-In T-Rex stable lines

To ensure low, near endogenous, levels of expression of recombinant proteins, stable cell lines that express PAK4, PAK5 and PAK6-GFP in a tetracycline inducible manner were generated following manufacturer's instructions (Invitrogen). U2OS-FlpIn-TRex cells were transfected with pcDNA5/FRT/TO-GFP vectors together with the plasmid pOG44 that encodes Flp recombinase. The expression of recombinant protein was induced with 0.5 to 1 µg/ml tetracycline for 24 h.

### Subcellular fractionation

U2OS cells stably expressing PAK4 wild-type and mutant for 14-3-3 binding sites were lysed using lysis buffer II (50 mM Tris HCl, pH 7.5, 0.5% Triton X-100, 0.15 M NaCl, 0.27 M sucrose) with protease inhibitors. Lysates were vortexed briefly and incubated for 3 min, followed by centrifugation at 16,000***g*** for 10 min at 4°C. Solubilised material was collected as the cytosolic fraction and the pellet as the insoluble actin bound fraction. The pellet was dissolved in an equal volume of SDS loading buffer and sonicated at 4°C. U2OS cells expressing PAK5 and PAK6 were lysed and subjected to cytoplasmic/membrane fractionation using a subcellular protein fractionation kit (Thermo Scientific) according to the manufacturer's instructions. Protein concentrations were determined using Coomassie Protein Assay reagent and equal amounts of protein were loaded onto 4–12% SDS-polyacrylamide gels.

### Mass spectrometry to identify phosphorylated residues

Immunoprecipitated proteins were alkylated with iodoacetamide and reduced using DTT before separation on precast gels. Gels were stained with Coomassie colloidal blue, proteins excised and digested in-gel with 5 µg/ml trypsin for 14 to 16 h. Tryptic peptides were analysed by LC–MS on ABI 4000 Q-TRAP system using precursor ion scanning in negative mode to search for the release of the [PO_3_]^−^ ion (−79 kDa) allowing ±1 Da [16260767], followed by MS/MS analysis in positive mode. The resulting data files were searched against the appropriate sequence, using Mascot run on an in-house server, with a peptide mass tolerance of 1.2 Da, fragment mass tolerance of 0.8 Da, and with variable modifications allowing phosphorylation of Ser/Thr or Tyr and methionine oxidation or dioxidation.

### Immunofluorescence and microscopy of fixed cells

Cells grown on coverslips were fixed with 3.7% paraformaldehyde, permeabilised with 0.2% Triton X-100, rinsed in PBS, stained with Phalloidin Alexafluor 594 and DAPI and mounted onto slides. For indirect staining, slides were permeabilised, incubated with blocking buffer (3% BSA in PBS), primary antibody overnight at 4°C followed by secondary antibody conjugated with Alexafluor 680. The slides were viewed under a Zeiss LSM710 microscope under a Plan-Apochromat 40× NA (numerical aperture) 1.3 oil immersion objective. Numbers of GFP-expressing cells were counted and those in which PAK4-GFP, PAK5-GFP and PAK6-GFP were colocalised with F-actin at the leading edge (A) or with β-catenin at cell–cell junctions (B and C) were scored as yes-or-no using merge channels and the JaCOP plugin of ImageJ [63], and *n* ≥ 60 per condition. Each sample was assessed 3 times and graphs in Figure 2 indicate means and standard deviations. The localisations were scored blind to the source of the signals.

### *In vitro* phosphorylation of bacterially purified group II PAK kinase-dead mutants

Bacterially expressed GST-PAK4 K350M S474A, GST-PAK5 K478M S602A and PAK6 K436M S560A (2 µg) were incubated with purified AGC kinases, 1 mM ATP and 100 mM MgCl_2_ at 30°C for 30 min. The reaction buffer contained 25 mM Tris–HCl pH 7.5 and 1 mM EDTA. Kinases were used at 1 U/µg of substrate, where one unit corresponds to the amount of protein kinase that catalyses the incorporation of 1 nmol of phosphate into the standard substrate in 1 min. Reactions were stopped with LDS sample buffer.

### Cell viability assay

Cells (2500 per well) were seeded in the wells of a 96-well plate and allowed to attach overnight. Cell viability was determined over a period of 96 h by using CellTiter 96® AQueous Non-Radioactive Cell Proliferation Assay kit (Promega) according to manufacturer's instructions. Results were plotted with the absorbance at 490 nm (±SD) on *Y*-axis versus time on *X*-axis.

### Wound healing assay

How the silencing of group II PAKs affected cell migration was tested in wound healing assays using IBIDI culture inserts (#80209) as per manufacturer's instructions. Approximately 7.5 × 10^4^ cells were seeded into the chambers to the left and right of the inserts. Cells were allowed to attach overnight. The insert was removed and the migration of cells into the 500 µm gap between the chambers was observed over a period of 15 to 24 h under a Nikon TIRF microscope with images taken every 2 min by a Photometrics cascade II CCD (charge-coupled device) camera using Nikon NIS Elements software.

### Transwell migration assay

Matrigel invasion assays were carried out using BD Biocoat^TM^ growth factor reduced Matrigel® Invasion Chambers (PET 8.0 µm, Corning) according to manufacturer's instructions. Briefly, cells were serum-starved for 2 h, detached using cell dissociation buffer (GIBCO), and 3 × 10^5^ cells in serum free medium were added to the upper chambers. Chemoattractant (medium containing 10% (v/v) FBS) was added to the lower wells. The chambers were placed at 37°C in 5% CO_2_ for 20 h. Non-invading cells in the upper chamber were scraped off and the migrated cells on the bottom of the chamber were fixed and stained using REASTAIN Quick-Diff kit (Gentaur Molecular Products) according to manufacturer's instructions.

### Lentiviral shRNA mediated gene silencing

The sequences of the lentiviral shRNAs targeting PAK4 and PAK6 from Sigma were:

shPAK4_1: CGGACTAAGAGGTGAACATGTATGCTCGAGCATACATGTTCACCTCTTAGTTTTTTG

shPAK4_2: CGGCGAGAATGTGGTGGAGATGTACTCGAGTACATCTCCACCACATTCTCGTTTTTG

shPAK6: CCGGGCACAGGGATATTTCTAAGAACTCGAGTTCTTAGAAATATCCCTGTGCTTTTT

To generate lentiviral particles, HEK293T cells were transfected with a plasmid mix containing MISSION pLK0.1-puro lentivirus plasmid (vector that encodes shRNA), pCMV delta R8.2 (packaging plasmid) and pCMV-VSV-G (pseudotyped envelope plasmid) using PEI. At 48 h post-transfection, the virus-containing medium was collected and filtered through a 0.45 µm surfactant-free, cellulose acetate filter (Sigma). SKMEL13 and SBcl2 melanoma cells were plated on a six-well plate, 24 h prior to viral infection. At 60% confluency, cells were infected with 2 ml per well of virus-containing medium with 10 µg/ml polybrene (Santa Cruz), and 48 h post infection, cells were selected in the presence of 1.5 µg/ml of puromycin.

### Reproducibility

All experiments, except mass spectrometry analyses and Figure 5C, were performed at least three times with significant reproducibility across replicates. Graphs were plotted and statistical significance assessed by ANOVA followed by Tukey's multiple comparison tests using GraphPad Prism 5.0; *P*-value ≤0.05 is considered statistically significant.

## Data Availability

The mass spectrometry proteomics data have been deposited to the ProteomeXchange Consortium via the PRIDE [64] partner repository with the dataset identifier PXD035415.

## Open Access

Open access for this article was enabled by the participation of University of Dundee in an all-inclusive *Read & Publish* agreement with Portland Press and the Biochemical Society under a transformative agreement with JISC.

## Abbreviations

AMPK: AMP-activated protein kinase
EGF: epidermal growth factor
FSK: forskolin
GEF: guanine nucleotide exchange factor
IGF1: insulin-like growth factor 1
PAK: p21-activated kinase
PKA: protein kinase A
PKB: protein kinase B (also known as Akt)
