# Supplementary material for: Differential roles and regulation of the protein kinases PAK4, PAK5 and PAK6 in melanoma cells
Source: Biochem J. 2022 Aug 31;479(16):1709–25. doi: 10.1042/BCJ20220184 (PMC9444074; doi:10.1042/BCJ20220184)
Supplement: Supplementary Material [file BCJ-479-1709-s1.pdf]

Supplementary figures and table

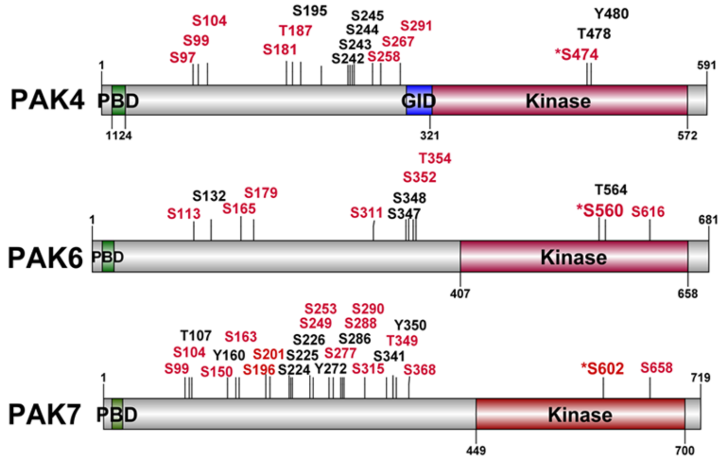

Figure S1

Schematic representation of the phosphorylation sites of PAK4, PAK5 and PAK6 identified by LC-MS/MS analysis (Orbitrap). The phosphorylation sites identified are indicated, the residues in red indicate the ones that could be assigned with confidence and those indicated by an asterisk are the activation loop serine residues.

A

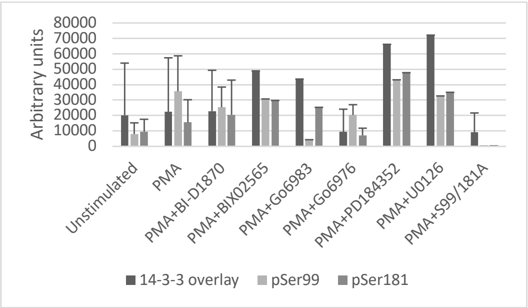

Arbitrary units
80000
70000
60000
50000
40000
30000
20000
10000
0
Unstimulated
PMA
PMA+BI-D1870
PMA+BIX02565
PMA+Go6983
PMA+Go6976
PMA+PD184352
PMA+U0126
PMA+S99/181A
14-3-3 overlay
pSer99
pSer181

B

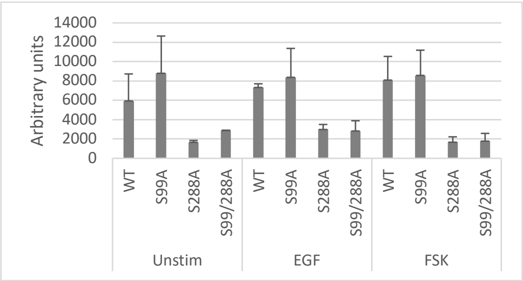

Arbitrary units
14000
12000
10000
8000
6000
4000
2000
0
WT
S99A
S288A
S99/288A
Unstim
EGF
FSK

C

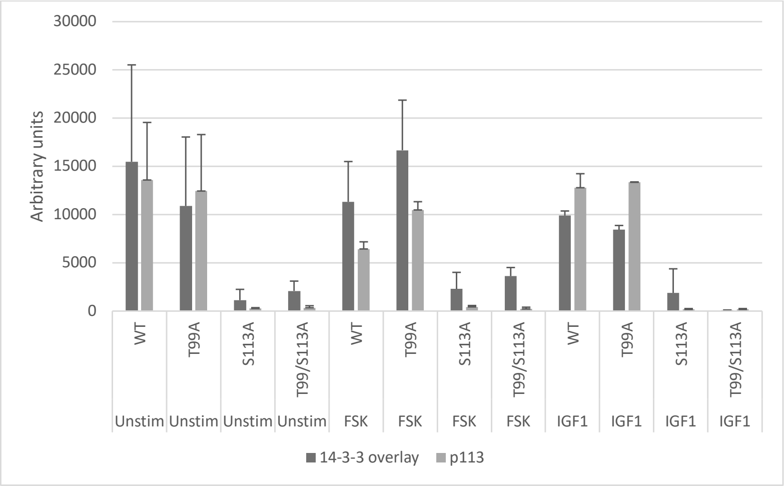

Arbitrary units
30000
25000
20000
15000
10000
5000
0
WT
T99A
S113A
T99/S113A
Unstim
FSK
IGF1
14-3-3 overlay
p113

D

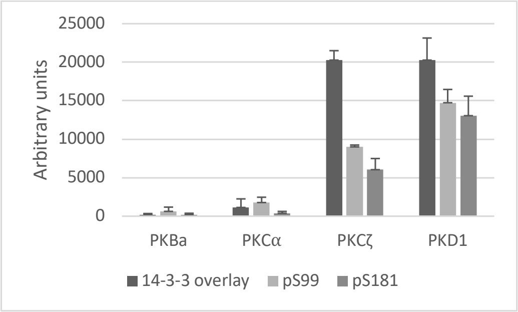

Arbitrary units
25000
20000
15000
10000
5000
0
PKBa
PKCα
PKCζ
PKD1
14-3-3 overlay
pS99
pS181

E

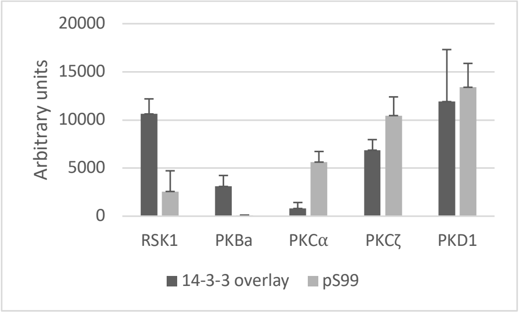

Arbitrary units
20000
15000
10000
5000
0
RSK1
PKBa
PKCα
PKCζ
PKD1
14-3-3 overlay
pS99

F

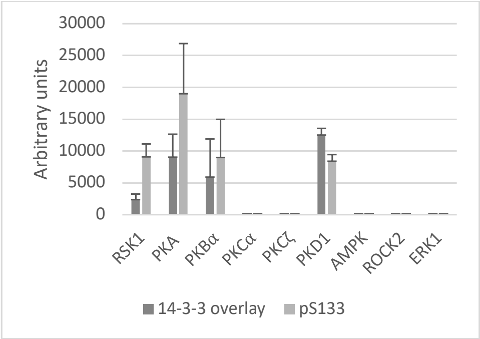

Arbitrary units
30000
25000
20000
15000
10000
5000
0
RSK1
PKA
PKBα
PKCα
PKCζ
PKD1
AMPK
ROCK2
ERK1
14-3-3 overlay
pS133

Figure S2

Although the quantitation and linearity range of 14-3-3 overlays have not been explored, and these should be considered as semi-quantitative, here ImageJ analyses (Schneider CA, Rasband WS, Eliceiri KW. (2012) NIH Image to ImageJ: 25 years of image analysis. Nat Methods. 9, 671-675) of the following results are displayed, each given for n=3 and indicating the standard deviation.

A. Image J analyses of data represented in Fig 2A. Gö6976 inhibits pSer181 phosphorylation and 14-3-3 binding to PAK4-GFP, whereas Gö6983 primarily inhibits phosphorylation of pSer99.

B. Image J analyses of data represented in Fig 2E. Mutation of Ser288 inhibits binding of PAK5-GFP to 14-3-3, assessed by 14-3-3 overlays.

C. ImageJ analyses of data represented in Fig 2H. Mutation of Ser113 inhibits binding of PAK6 K436M S560A-GFP to 14-3-3 in 14-3-3 overlays.

D. ImageJ analyses of data represented in Fig 2C showing data for protein kinases that phosphorylate Ser99 of GST-PAK4 K350M S474A.

E. ImageJ analyses of data represented in Fig 2F showing data for protein kinases that phosphorylate Ser99 of GST-PAK5 K478M S602A.

F. ImageJ analyses of data represented in Fig 2I showing data for protein kinases that phosphorylate Ser133 of GST-PAK6 K436M S560A.

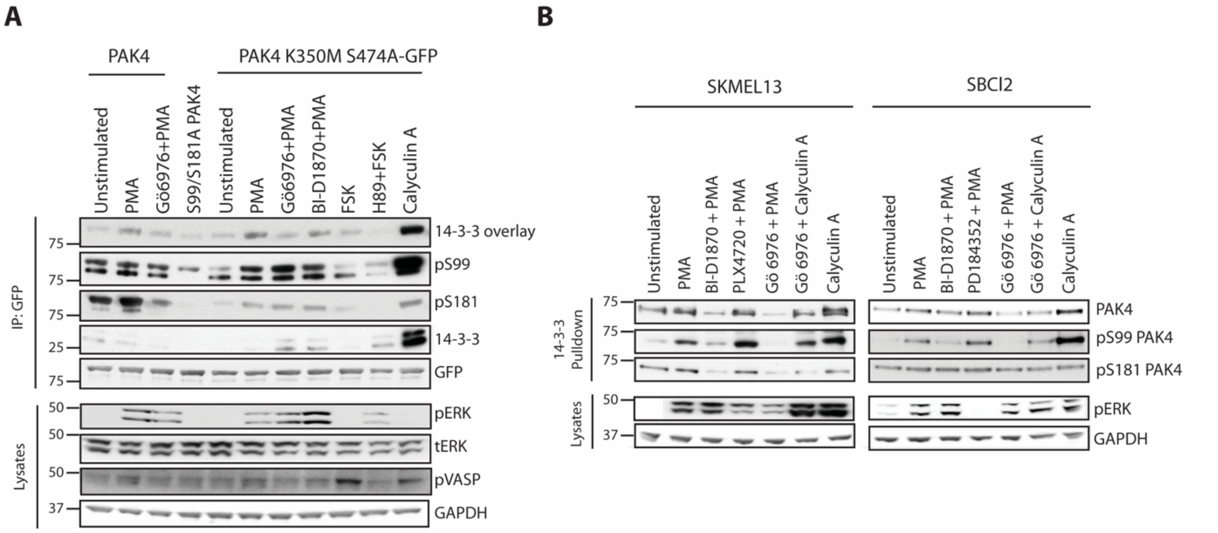

Figure S3

A. GFP-tagged PAK4 K350M S474A was immunoprecipitated from cells treated with kinase activators and inhibitors as indicated and binding to 14-3-3s was analysed. Phosphorylation state of Ser99 and Ser181 was analysed using phosphospecific antibodies. Cell lysates (30 µg) were blotted for pThr202/Tyr204 ERK, pSer473 PKB and pS157 VASP.

B. SKMEL13 and SBcl2 melanoma cells were serum starved overnight and treated with MAPK and PKC/PKD inhibitors as indicated. Cell lysates from each condition were pre-cleared, incubated with 14-3-3 Sepharose beads overnight and eluted in sample buffer. The eluates were subjected to SDS-PAGE and blotted for PAK4. The phosphorylation state of PAK4 was analysed using pSer99 and pSer181 antibodies. Cell lysates (30 µg) were analysed with antibodies that recognise PAK4, pThr202/Tyr204 ERK and GAPDH.

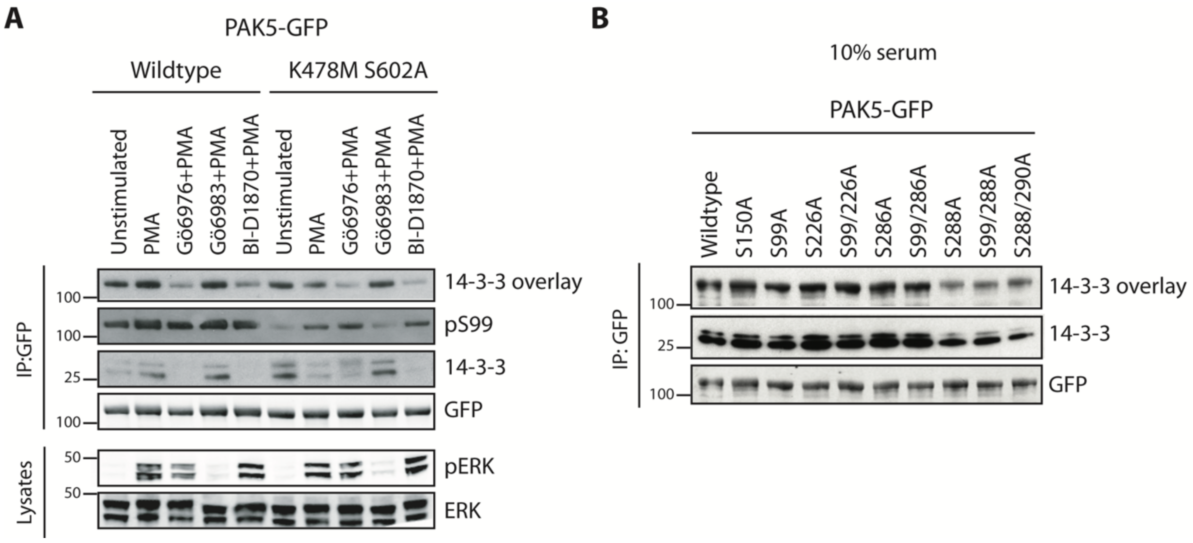

Figure S4

A. PAK5-GFP was immunoprecipitated from lysates of cells serum starved overnight and treated with the combination of kinase activators and inhibitors as indicated. The immunoprecipitates were tested for binding to 14-3-3s.

B. Cell lysates from HEK293 cells over expressing PAK5 wildtype and mutant for predicted 14-3-3 binding sites were immunoprecipitated and tested for their ability to bind directly to 14-3-3s in Far-Western overlay assay and by co-immunoprecipitation of endogenous 14-3-3s

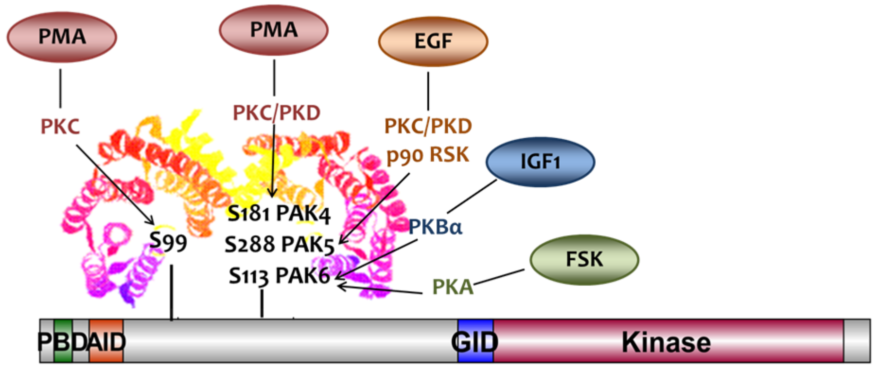

Figure S5

Map of the 14-3-3 binding sites of group II PAK kinases showing the stimuli and kinases regulating phosphorylation of these sites.

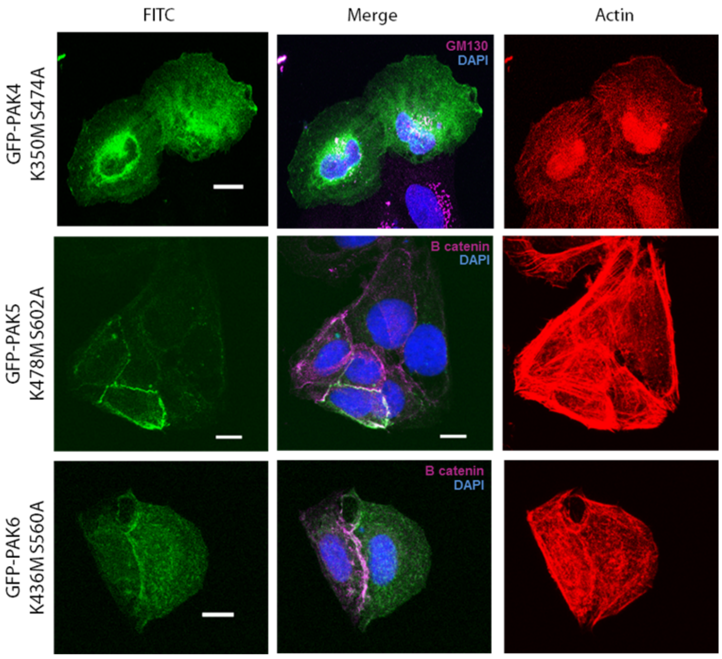

Figure S6

U2OS cells were transfected with PAK4, PAK5 and PAK6 kinase-dead mutants. The cells were seeded onto coverslips 16 h post-transfection and allowed to attach overnight. Cells were fixed in paraformaldehyde, stained for actin (Alexafluor 594 Phalloidin), nucleus (DAPI) and mounted onto slides. The samples were imaged using LSM710 confocal microscopy; scale bar = 20 µm.

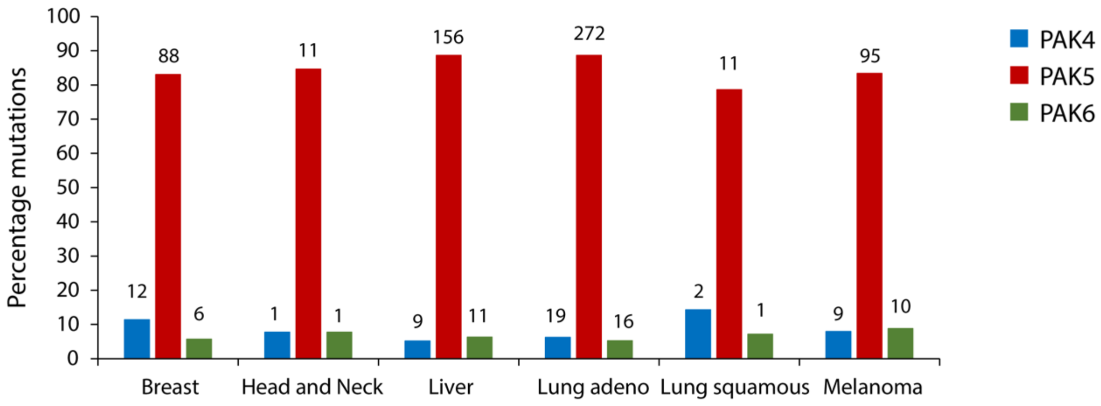

Figure S7

Graph representing the number of mutations carried by PAK4, PAK5 and PAK6 in various cancers (data from Alexandrov et al., 2013 Signatures of mutational processes in human cancer. Nature. 500, 415-421. (23)).

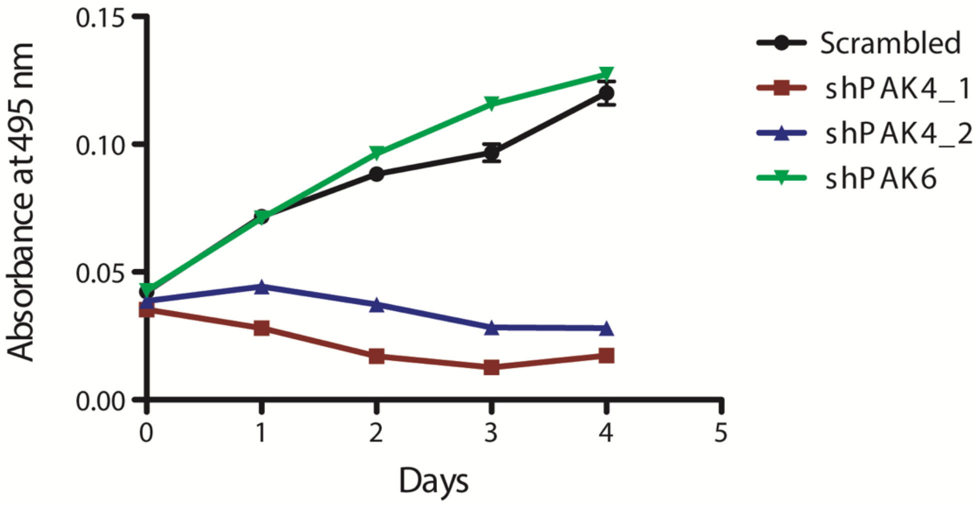

Figure S8

PAK4 and PAK6 knockdown SBcl2 cells were seeded in 96-well plates and cell proliferation was monitored over a period of 96 h using CellTiter AQueous non-radioactive cell proliferation assay kit.

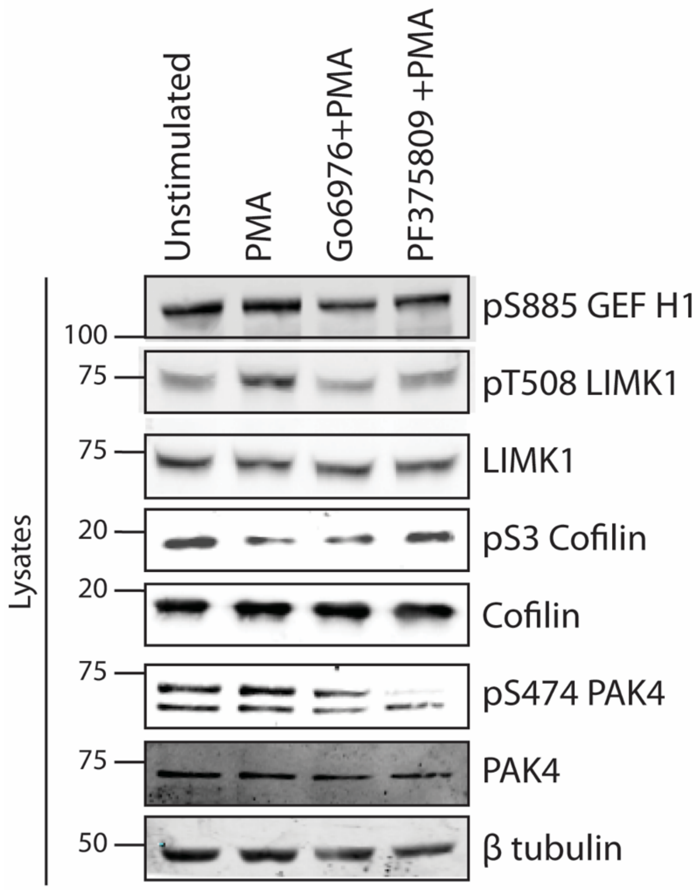

Figure S9

Lysates from SKMEL13 cells, serum-starved overnight and pre-treated with Gö6976 and PF-3758309 followed by PMA, were analysed for phosphorylation of GEF H1, LIMK1 and cofilin with β-tubulin as the loading control.

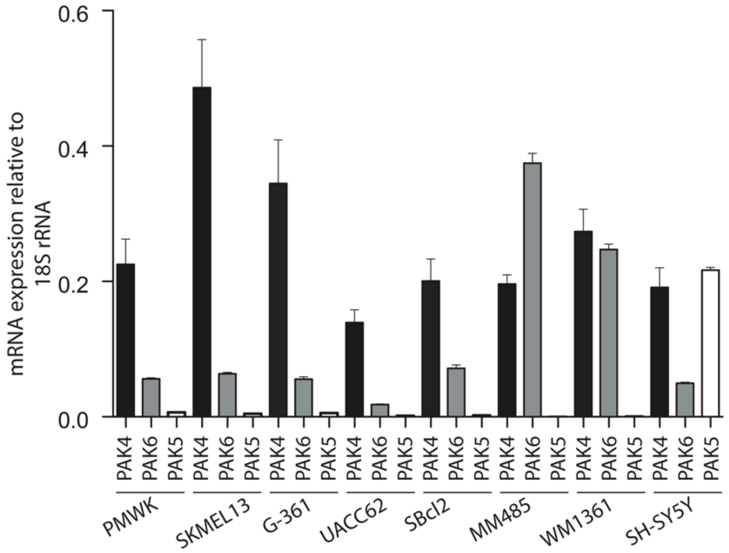

Figure S10

The mRNA expression profile of group II PAK kinases in various melanoma cell lines carrying B-RAF V600E (SKMEL13, UACC62 and G361) and N-RAS Q61K (SBcl2, WM1361, MM485) driver mutations relative to 18S rRNA levels are represented. The mRNA from SH-SY5Y cell line (neuroblastoma) was used as the positive control for PAK5 expression.

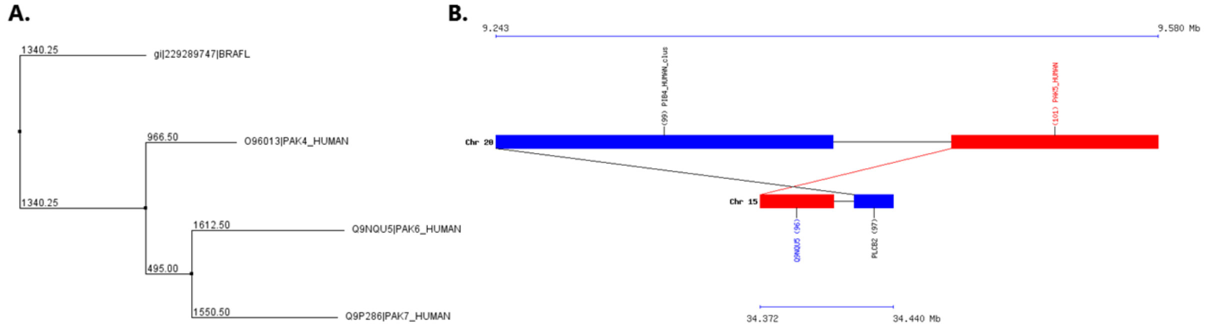

Figure S11

A. Phylogenetic tree of group II PAK kinases with *Branchiostoma* PAK protein as the root constructed using Jalview with PAM250 substitution matrix.

B. Chromosome plot of PAK5 gene (in red on paralagon on Chromosome 20) and PAK6 gene (Q9NQU5, on paralagon on Chromosome 15) (http://wolfe.ucd.ie/dup/human5.28/).

Table S1. Phosphorylation sites of *in vitro* phosphorylated PAK5 K478M S602A (Orbitrap data)

**Kinase**	**Phosphopeptides**	**Potential residues**
RSK1	R.SGsGLQEPMMPFGASAFK.T<br>R.SRsGSGLQEPMMPFGASAFK.T.<br>R.SRSGSGLQEPMMPFGASAFK.T<br>K.IGEGSTGIVCIATEKHTGKQVAVMKMDLR.K	Ser288/290<br>Ser286/288/290<br>Ser286/288/290<br>Ser459/Thr460/467/471
PKA	No significant phosphopeptides detected	
PKBα	R.SGsGLQEPMMPFGASAFK.<br>R.SRSGSGLQEPMMPFGASAFK.T<br>R.SRsGSGLQEPMMPFGASAFK.T	Ser288/290<br>Ser286/288/290<br>Ser286/288/290
PKCα	R.ASSSsPLDYSFQFTPSR.T<br>K.SSYLNQTSPQPtMRQR.S	Ser223/224/225/226<br>Thr281
PKCζ	R.ASSSsPLDYSFQFTPSR.T	Ser223/224/225/226/231/<br>Tyr230
PKD1	K.IEISGPsNFEHR.V<br>K.KIEISGPsNFEHR.V<br>R.DIKSDSILLTSDGR.I<br>K.ALVGTPyWMAPEVISR.L<br>K.THPQGHSYNSYTYPR.L<br>R.AQMVLsPPLsGSDTYPR.G<br>R.ASSSSPLDYSFQFTPSR.T<br>K.HGEAYYSEVKPLKsDFAR.F<br>R.SRSGSGLQEPMMPFGASAFK.T<br>K.ESLAYSEsEWGPSLDDYDR.R<br>R.FSADYHSHLDSLSKPSEYSDLKWEYQR.A	Ser11/14<br>Ser14<br>Ser571/573/Thr577<br>Thr606/Tyr608<br>Thr306/Ser312/Tyr313<br>Ser341/345<br>Ser223/224/225/226<br>Ser183/190<br>Ser286/288/290<br>Ser253/255<br>Ser196/201/205/207/210/21<br>3
AMPK	K.ALVGtPYWMAPEVISR.L	Thr606/Tyr608
ROCK II	R.FSADYHSHLDSLSKPSEYSDLKWEYQR.A	Ser196/201/205/207, Tyr199
ERK1	K.SSYLNQTsPQPTMR.Q<br>K.ALVGtPYWMAPEVISR.L<br>R.AQMVLsPPLSGSDTYPR.G<br>R.AQMVLsPPLsGSDTYPR.G<br>R.ASSSSPLDYSFQFTPSR.T<br>R.ASSSSPLDYSFQFTPSR.T<br><br>R.RPKSSYLNQTsPQPTMR.Q<br>K.ESPPTPDQGASSHGPGHAEENGFITFSQYSSES<br>DTTADYTTEK.Y	Thr276/Ser277<br>Thr606<br>Ser341<br>Ser341/345/347<br>Thr235/Ser237<br>Ser223/224/225/226/231/23<br>6/ Thr234<br>Ser270/271/277/Thr276/281<br>Ser104/113/114/129/Thr107
